# Solid-State Fermentation Engineering of Traditional Chinese Fermented Food

**DOI:** 10.3390/foods13183003

**Published:** 2024-09-22

**Authors:** Guangyuan Jin, Yujie Zhao, Shuhan Xin, Tianyi Li, Yan Xu

**Affiliations:** Lab of Brewing Microbiology and Applied Enzymology, School of Biotechnology, Jiangnan University, Wuxi 214122, China; g.jin@jiangnan.edu.cn (G.J.); 6210208185@stu.jiangnan.edu.cn (Y.Z.);

**Keywords:** solid-state fermentation, traditional Chinese fermented foods, process engineering, artificial intelligent

## Abstract

Solid-state fermentation (SSF) system involves solid, liquid, and gas phases, characterized by complex mass and heat transfer mechanisms and microbial complex interactions. The SSF processes for traditional Chinese fermented foods, such as vinegar, soy sauce, and baijiu primarily rely on experience, and most of the operations are replaced by auto machine now. However, there is still a lack of engineering in-depth study of the microbial process of SSF for complete process control. To meet the demands of smart manufacturing and green production, this paper emphasizes the engineering analysis of the mechanisms behind SSF. It reviews the progress in the engineering aspects of Chinese traditional SSF, including raw material pretreatment, process parameter detection, mathematical model construction, and equipment innovation. Additionally, it summarizes the challenges faced during intelligent upgrades and the opportunities brought by scientific and technological advancements, proposing future development directions. This review provides an overview of the SSF engineering aspects, offering a reference for the intelligent transformation and sustainable development of the Chinese traditional SSF food industry.

## 1. Introduction

**Figure 1:**
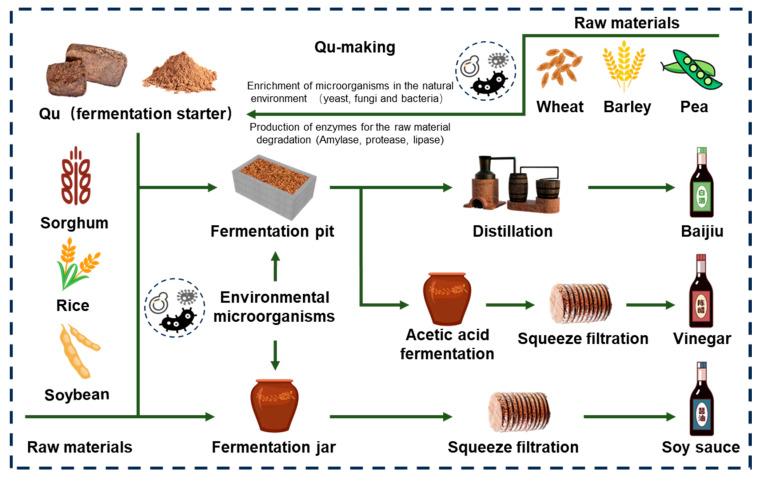
Schematic diagram of the production process of traditional Chinese SSF foods (Baijiu, vinegar, soy sauce).

Solid-state fermentation (SSF) refers to a microbial fermentation process that takes place in a solid culture medium that contains rich water but little or no free water. It includes mixed strains, in which the substrate is a water-insoluble polymer, which not only provides the functional microorganisms with the required carbon source, nitrogen source, inorganic salts, water and other influencing substances, but also serves as a place for microbial growth [1,2]. China has a very long history of SSF, especially in the field of food and beverage including vinegar, baijiu and soy sauce (Figure 1). It is not only widely loved by people and has become a part of daily life, but has also increasingly become a cultural name card in the Chinese food industry and has attracted the attention of the international community [2].

The traditional Chinese fermented food industry is still dominated by semi-manual and semi-mechanized processes, complex microbiota and natural fermentation [3], except for the initial matrix composition, there is basically no process parameter control. This empirical production method is inefficient and energy-intensive, with unstable product quality and is easily affected by environmental and human factors. Because it is a solid matrix, the entire fermentation system is in a non-homogeneous state, and the mass and heat transfer mechanisms between the solid, liquid and gas phases are complex, which makes it difficult to monitor and control relevant parameters in the fermentation process, and the mechanized and intelligent transformation and upgrading of the industry faces challenges.

At present, the research on traditional SSF is mostly concentrated in the fields of microbiology and flavor chemistry, and there are few reports on research in the field of engineering. Traditional SSF should conform to the current development status of society, green production, intelligent manufacturing, and the important thing is to analyze the mechanism of SSF from an engineering perspective. Coupled with the improvement and innovation of equipment, the process parameters are optimized and the production process is highly mechanized, which ultimately promotes the stable and high-quality development of traditional industry. This article uses the traditional food industry as a medium to briefly introduce the current situation, problems, research methods and progress of related engineering in traditional SSF, in order to provide a reference for promoting the stable and high-quality development of traditional Chinese SSF food industry.

## 2. Current Status of Traditional SSF Food Production

### 2.1. Vinegar

The traditional SSF process of Chinese vinegar mainly includes four stages: Qu-making, starch saccharification and ethanol fermentation, acetic acid fermentation, and aging. Qu-making and acetic acid fermentation are aerobic fermentation, while ethanol fermentation is anaerobic fermentation, which is also the key process of flavor formation [4]. Chinese vinegar has a strong regional attribute, products from different regions have their own unique flavor characteristics, such as the well-known Shanxi Aged Vinegar, Zhenjiang Aromatic Vinegar, Sichuan Bran Vinegar, Shanghai Rice Vinegar, Jiangsu and Zhejiang Rose Vinegar, Fujian Red Rice Vinegar [5].

Chinese vinegar enterprises still face challenges in production methods. The industry concentration is low. The SSF stage still uses traditional process flow, relying on the experience of technical personnel to guide key links. There is a lack of unified quantitative standards, and the level of industrialization needs to be improved. Although most vinegar companies still rely on manual or semi-manual production methods, mechanized equipment is also being put into vinegar production. The traditional manual and mechanized process flows are the same, but the process implementation methods are optimized. For example, the vertical and horizontal fermentation tanks designed to replace the traditional fermentation jar mainly solve the problem of mechanical turning over to replace manual turning over [6]. This also indirectly shows that the technology level of the vinegar industry has a large span. Even the traditional manual production process must obey the management of modern production to ensure product quality and food safety. After introducing mechanical equipment, a few large enterprises are faced with the problem that the flavor is different from the traditional taste, which directly restricts the improvement of the production efficiency and production scale of vinegar enterprises [7]. Improving the SSF control technology has a far-reaching impact on the quality and scale of the industry.

### 2.2. Soy Sauce

Compared to the vinegar industry, some large soy sauce companies have a higher degree of mechanization. Japanese soy sauce technology is in the leading position, most are produced in large modern factories with computer-controlled technology [8]. At present, Chinese soy sauce manufacturing has entered the early stage of an automated and digitally controlled production to a greater or lesser extent, some large companies are at the forefront [9].

The two typical production processes for soy sauce fermentation are high-salt dilute fermentation and low-salt SSF [10]. High-salt dilute fermentation includes Cantonese method and Japanese method. High-salt dilute fermentation has a long cycle, generally 3–6 months, and has high requirements for fermentation operators and raw materials. The soy sauce produced is of high quality and rich in flavor [11]. Low-salt SSF has a short cycle, generally 20–30 days, which is simple and efficient, with good economic benefits, but the taste is not as rich as the soy sauce produced by high-salt dilute fermentation. From the perspective of market share, high-salt dilute soy sauce has become the mainstream, but high-salt dilute fermentation also faces problems such as high production cost and long fermentation cycle, low-salt solid soy sauce still has a place [12]. Therefore, it is necessary to further analyze the mechanism of traditional process, explore the improvement method of fermentation process, effectively combine traditional process with modern process, and improve the brewing technology and product quality of soy sauce.

### 2.3. Baijiu

**Figure 2:**
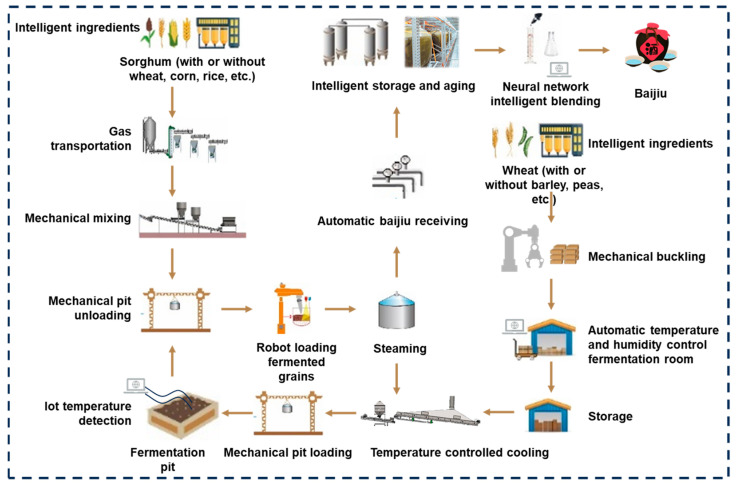
Current status of traditional SSF food production (taking Chinese Baijiu as an example). Reprinted/adapted with permission from Ref. [13] (2024, *Food and Fermentation Industries*).

In recent years, the production process of Chinese Baijiu has been significantly improved. The introduction of modern technology has optimized the traditional process. From raw materials to products, each link has achieved different degrees of mechanization and process modernization (Figure 2) [13]. First, in terms of raw material processing, the widespread use of modern equipment such as high-efficiency grinders and cooking equipment has improved raw material utilization and saccharification efficiency. The use of more scientific and modern gas transmission pipelines combined with near-infrared spectroscopy technology for online monitoring has provided equipment and data foundation for intelligent grain distribution [13]. During the Daqu fermentation, the treading and turning of the Daqu has also been replaced by machines, reducing labor intensity and improving the working environment [14]. In addition, combined with the Internet of Things technology, not only the temperature and humidity in the fermentation room can be monitored online, but also the temperature in the fermentation pit, providing data support for optimizing the Daqu fermentation and the parameters of pit loading [15]. The analysis and improvement of distillation technology is another important aspect [16]. It not only reduces energy and water consumption, but also reduces the impact of human factors through automatic classification and Baijiu receiving, thus improving product stability. In terms of storage and aging, the combination of stainless steel tanks and ceramic jars makes the aging process more scientific, and the stability and quality of the Baijiu are guaranteed [17].

Although the production of Baijiu has undergone a significant mechanization upgrade compared to traditional manual production, when it comes to the SSF mechanism, production guidance still relies more on experience. In particular, in the Daqu fermentation process, including the shaping of the Daqu and the process in the fermentation room, there are still differences in the bacterial community structure [18] and flavor substances [19] between mechanized and manual, resulting in a decline in quality. The unclear mechanism of the Daqu fermentation process has led to the fact that Daqu production is still mainly based on traditional fermentation room, which is labor-intensive and the product is prone to fluctuations. Therefore, the analysis of the engineering mechanism of Daqu SSF is the basis for the mechanized and intelligent transformation of Daqu, and is also the key to promoting the upgrading of the Baijiu industry [20].

## 3. SSF Process Engineering Study

### 3.1. Raw Material

Traditional solid-state fermented food raw materials are mainly starch and protein, such as sorghum, wheat and soybean (Table 1). Compared to submerged fermentation, the traditional SSF uses a simultaneous saccharification and fermentation process. This may cause an in-sufficient saccharification, but it is necessary to slow-down the fermentation process to prevent the heat accumulation and flavor formation. Still, a certain pretreatment of raw material, for example steam cook, is necessary to improve the enzyme (including glucoamylase, α-amylase, protease, etc.) efficiency saccharification. The raw materials are pretreated according to the required products and the corresponding fermentation process to improve product quality.

### 3.2. Process Parameters

The Baijiu Jiupei is fermented while saccharifying. The raw material cooking process has a direct impact on the saccharification rate, which in turn affects the entire fermentation process. For example, Zhao et al. [21] used steam explosion technology to destroy the starch structure of sorghum, improve saccharification efficiency and ethanol yield, and reduce waste in the liquor industry. Although the actual production effect needs to be combined with the sensory evaluation of subsequent fermentation products, it also provides ideas for the regulation of Baijiu fermentation. Yan et al. [22] found that after electron beam irradiation of sorghum, the internal structure of starch granules was severely damaged, the fermentation efficiency was improved, and the ethanol production increased by 43.44%. The waxy layer on the surface of sorghum reduces the hydrolysis efficiency of starch. By developing a sorghum dewaxing process [23], the ethanol yield of sorghum fermentation has been increased (Table 1). The extracted wax can be used as a value-added by-product to improve economic benefits [24]. To promote the industrialization of Tibetan highland barley Baijiu, four barley processing methods, namely, stir-frying, microwave, crushing, and stir-frying with crushing, were evaluated from the three perspectives of ethanol concentration of fermented grains, flavor substances, and sensory evaluation. The results showed that stir-frying with crushing helped improve the quality of highland barley Baijiu [25]. Before SSF, wheat was microwaved (700 W, 30 s), which increased the production of saccharifying enzymes by 60% and protease by 25% [26]. In addition, microwave treatment helped to store raw materials and reduce fungal infection [27].

Soybeans are the main protein raw material for soy sauce. In the traditional production process, soybean pretreatment is completed through soaking and steaming, which is not only energy-consuming and water-consuming, but also limits the expansion of industrial scale [28]. Therefore, new soybean pretreatment processes have attracted extensive research (Table 1). Zhang et al. [29] used extrusion puffing technology to treat soybean in the process of brewing soy sauce. The results showed that this method increased the volatile compounds of soy sauce, improved the flavor, and the protein utilization rate reached 89.56%, which was higher than the steaming process. Zhang et al. [30] studied and optimized the soybean microwave vacuum pretreatment process. Compared with the traditional steaming process, the total amino acid content in the finished Qu was significantly increased. Zhang et al. [31] used steam explosion technology to destroy the polysaccharide and protein structure of soybeans, shortened the fermentation time of soy sauce, and improved the soybean fermentation utilization rate and soy sauce quality.

**Table 1:** Raw material pretreatment methods.

Raw Material	Pretreatment	Effect	Reference
Sorghum	Steam explosion	Ethanol yield:20.5 g/100 g	[21]
	Electron beam irradiation	Ethanol production increased by 43.44%	[22]
	Dewaxing	Ethanol yield: 117.8 g/L, an increase of 36.8%	[23]
Barley	Stir-fry with crush	Ethanol content of fermented grains: 18.5%vol	[25]
Wheat	Microwave	Glucoamylase production increased by 60%, protease production increased by 25%	[26]
Soybean	Extrusion puffing	Protein utilization rate: 89.56%	[29]
	Microwave vacuum drying	Neutral protease activity increased by 18.79%, acidic protease activity increased by 35.77%	[30]
	Steam explosion	Cellulose was degraded by 50.7%, hemicellulose was degraded by 71.1%	[31]

SSF process parameters are key indicators reflecting the state of the fermentation process. How to quickly and accurately monitor and control them is the difficulty and key point in realizing the engineering scale-up, regulation and optimization of the SSF process.

#### 3.2.1. Moisture

Although SSF has almost no free-flowing water, the moisture of the substrate is still an important parameter of the fermentation process. Too high or too low a moisture will cause the fermentation process to be abnormal. Therefore, it is particularly necessary to quickly detect the moisture during the fermentation process to ensure that the substrate moisture is in an ideal state [32].

The constant weight drying method commonly used for moisture detection is time-consuming. Rapid infrared moisture detectors based on the same principle shorten the detection time, but still cause damage to the substrate [33]. In order to achieve rapid and non-destructive online monitoring of moisture, the methods currently reported in exploratory research include nuclear magnetic resonance [32,34,35], near infrared spectroscopy [36,37] and hyperspectral imaging [38,39,40]. The systems involved include Daqu [32], soybean meal fermentation [37], Cupei [40], barley fermentation [34], and pit mud [39], as shown in Table 2.

Traditional offline detection has a high delay and cannot achieve the purpose of real-time monitoring and control. Nuclear magnetic resonance technology can help to achieve real-time monitoring of moisture in SSF, analyze moisture movement from a microscopic perspective, and analyze the fermentation process and the change law of moisture content [32]. Near-infrared spectroscopy technology can be quickly measured, low cost, and simple to operate, but there may be problems such as difficulty in signal extraction and low sensitivity. Hyperspectral imaging technology is based on the fast and non-destructive characteristics of near-infrared spectroscopy technology. When combined with imaging technology, it can achieve high-precision monitoring and distribution visualization of moisture, but the data volume is large, information redundancy increases, and equipment requirements are increased.

#### 3.2.2. Temperature

Fermentation temperature is closely related to the growth and metabolic state of microorganisms in the fermentation system and directly affects the final fermentation results [41]. Zhang et al. [42] found that changing the initial temperature would lead to differences in microbial succession and flavor substances during the fermentation of Jiangxiangxing Baijiu, revealing the importance of the initial temperature of the SSF process. Understanding the heat change mechanism of the SSF process and real-time temperature monitoring are of great significance for the optimization of fermentation process regulation and mechanical automation. At present, the temperature of Baijiu fermentation process is obtained by manual collection. The insertion depth and position of the thermometer are easily affected by human factors, which leads to increased data error. In addition, the workload is large and the data is not intuitive and lacks continuity.

Real-time temperature monitoring and selection of appropriate temperature control methods are the prerequisites for regulating fermentation temperature. Zhang et al. [15] proposed a real-time monitoring system for fermentation pit temperature based on Zigbee technology. Li et al. [43] designed a pit temperature visualization system combining temperature acquisition module and data visualization module. Pitol et al. [44] collected the average temperature change data of different horizontal positions of the matrix during the fermentation process by installing a thermocouple sensor combination in the packed bed bioreactor. Fan et al. [45] designed an intelligent temperature control system based on temperature sensors, realizing real-time monitoring of the temperature of Cupei at different stages, providing a scientific basis for optimizing the vinegar fermentation process in different seasons. The results show that combining sensor technology with wireless communication technology helps to realize temperature monitoring automation and data visualization, and can intuitively understand the temperature changes and fermentation trends in the substrate. However, the results are closely related to sensor factors. For example, sensor sensitivity directly affects data accuracy.

#### 3.2.3. Acidity

The acidity during SSF has an important influence on the evolution of microbial communities and the production of metabolites. For example, in Baijiu fermentation, appropriate acidity not only helps the gelatinization and saccharification of starch and inhibits bacteria, but also helps esterification, which serves as a flavoring substance in the liquor [46]. The traditional chemical acid-base titration method and pH meter method are cumbersome to operate, have high latency, and cause substrate loss and environmental pollution, making it difficult to achieve large-scale rapid monitoring [47]. In order to find a fast, non-destructive and clean acidity monitoring method to meet the needs of information monitoring, the methods currently reported include monitoring systems based on Zigbee wireless communication technology, near-infrared spectroscopy technology and hyperspectral imaging technology (Table 2).

Xiao et al. [48] designed an online monitoring system for pH of fermentation pit based on Zigbee wireless communication technology. By comparing the manual sampling data, they realized the real-time online monitoring of pH changes in the fermentation pit. Compared with traditional manual sampling, it has the advantages of continuity and simple operation process, which promotes the automation process of SSF parameter monitoring. In addition to being used for moisture monitoring, near-infrared spectroscopy and hyperspectral imaging can also be used to detect the acidity of the fermentation system efficiently and quickly. Dai et al. [37] used near-infrared spectroscopy to quantitatively analyze the acidity of soybean meal fermentation, realized real-time monitoring of SSF process parameters. By establishing and optimizing the analysis model (R^2^ = 0.9433), they achieved rapid detection of Jiupei acidity. In the same Baijiu Jiupei system, Jiang et al. [49] applied hyperspectral imaging technology to establish a least squares support vector machine model, which further improved the detection accuracy of acidity (R^2^ = 0.9988). Xu et al. [50] achieved rapid detection of total acid content in broad bean SSF process based on near-infrared spectroscopy (R^2^ = 0.917). The development of online monitoring system can promote the intelligent upgrade of traditional SSF process monitoring and control.

#### 3.2.4. Porosity

The porosity of the matrix is an important parameter for studying mass transfer and microbial strains in SSF. Porosity is also an important physical property of the fermentation matrix. Although a higher porosity is beneficial for improving gas exchange and heat transfer and providing sufficient growth space for the microbial, it is also necessary to consider factors such as the nutritional needs of the microbial and the structure of the matrix bed and make a compromise [51,52,53]. Porosity is the volume fraction of voids in a porous matrix. Currently, the most commonly used methods for porosity detection are volume analysis and gravimetric analysis [54]. Casciatori et al. [53] used gravimetric analysis to evaluate the porosity of mixed substrate of sugarcane bagasse and wheat bran in a packed bed bioreactor and found that the porosity decreased almost exponentially with the increase of moisture content. Jiang et al. [55] used volumetric analysis to evaluate the effect of substrate porosity on flavor substances. They found that as the amount of rice husk added increased (0%–40%), the porosity of the substrate increased, and the most abundant flavor substances were found when the porosity was 32.53%. Volumetric analysis and gravimetric analysis can provide information about the average porosity of the substrate. Based on this method, a function between porosity, moisture content and biomass change can be established to predict the overall average porosity change of the fermentation system [54]. However, they cannot express the microscopic pore distribution.

In addition to volumetric analysis and gravimetric analysis, some studies have reported methods that combine digital image processing technology to evaluate the porosity of SSF substrate. Canedo et al. [56] used digital photography and X-ray tomography to obtain the 2D and 3D structures of the mixed substrate of sugarcane bagasse and wheat bran, respectively. The porosity was estimated by digital image processing technology, the results obtained by 2D digital photography and μ-CT were similar. Yancy-Caballero et al. [57] used X-ray microtomography to construct a pore network model and analyzed the connectivity inside bagasse particles from a microscopic perspective. 2D photography technology is simple to use and can provide the overall bed structure, but it is difficult to distinguish whether the pores are located on the upper or lower surface and whether the substrate particles and hyphae are located. 3D technology is more complex and can express the spatial distribution of pores and the microstructural characteristics of the substrate. Combining digital image processing analysis with porosity can help improve and optimize the heat and mass transfer model in the fermentation substrate.

#### 3.2.5. Biomass

Analyzing the structure of microbial communities is a prerequisite for controlling traditional food fermentation [58]. However, the SSF system is complex and microbial quantification is very difficult [59], especially for on-line detection. Indirect estimation methods based on specific microbial components include the glucosamine content method [60], the ergosterol method [61], the Kjeldahl nitrogen determination method [62], and the nucleic acid method [63]. However, these methods are complex and time-consuming. The blood cell plate count method sometime requires the preparation of a spore suspension before measurement (for mold), which is more troublesome and the results are easily affected by subjective factors [64].

Zheng et al. [65] achieved rapid detection of *Bacillus subtilis* in the SSF process of rapeseed meal by near-infrared spectroscopy. However, near-infrared spectroscopy technology is relatively expensive and has difficulties in measuring samples with large surface areas. With the development of sensor technology, the method of indirectly estimating biomass through oxygen uptake rate and carbon dioxide escape rate has been widely used. By analyzing the changes in O_2_ concentrations in the exhaust gas and using the logistic model to establish the correlation between oxygen uptake rate and biomass concentration, it is helpful to monitor the biomass concentration of the fermentation system in real time [64,66]. Compared with other methods, the results of this method are more accurate. Digital image processing technology has also been applied to the research on detecting biomass. By photographing the substrate and combining image processing with kinetic models, the changes in biomass in the fermentation system can be estimated [67,68,69]. However, digital image processing technology only targets surface phenomena and does not consider the microbial factors inside the substrate.

**Table 2:** Application of new technology for detecting parameters in SSF process.

Parameter	Method	Substrate	Reference
Moisture	Nuclear magnetic resonance	Wheat	[32]
		Glutinous rice	[35]
		Barley	[34]
	Near infrared spectroscopy	Wheat bran	[36]
		Soybean meal	[37]
	Hyperspectral imaging	Daqu	[38]
		Cupei	[40]
		Pit mud	[39]
Temperature	Temperature online monitoring system based on Zigbee	Jiupei	[15]
	Temperature visualization system based on embedded technology and wireless sensor network technology	Jiupei	[43]
	Multi-channel temperature acquisition module	Cupei	[45]
	Thermocouple temperature acquisition sensor combination	Wheat bran and sugarcane bagasse	[44]
Acidity	Acidity online monitoring system based on Zigbee	Jiupei	[48]
	Near infrared spectroscopy	Soybean meal	[37]
		Broad bean	[50]
	Hyperspectral imaging	Jiupei	[49]
		Cupei	[70]
Porosity	Digital image processing	Wheat bran and sugarcane bagasse	[56]
	X-ray tomography	Sugarcane bagasse	[56,57]
Biomass	Near infrared spectroscopy	Rapeseed meal	[65]
	Oxygen uptake rate and carbon dioxide release rate monitoring model	Wheat bran and linseed oil cake	[66]
		Wheat bran	[64]
	Digital image processing	Potato dextrose agar	[67]
		Wheat straw	[68]
		Sugarcane bagasse	[69]

### 3.3. Mathematical Model and Equipment Innovation

The traditional SSF process is difficult to control, the semi-manual and semi-mechanized production method leads to fluctuations in product quality. By establishing a SSF process engineering mathematical model, we can better understand the traditional process, lay the foundation for future process optimization and equipment design, and promote the mechanization transformation process.

#### 3.3.1. Mathematical Model

Jin et al. [71] developed a mathematical model for anaerobic SSF based on the Han-Levenspiel product inhibition equation. The model described that the heat generated during the traditional Chinese Baijiu fermentation process is mainly related to ethanol production, and the soil temperature around the fermentation jar plays a key role in the heat dissipation of the fermentation system. The model can predict whether the system will overheat during scale-up and production at different soil temperatures, which is helpful to improve traditional anaerobic SSF.

Packed bed bioreactors are prone to overheating when they are scaled up. Perez et al. [72] used a two-phase two-dimensional model to predict the maximum temperature and maximum bed height of the packed bed bioreactor, proving that the packed bed bioreactor scale-up theory is feasible. However, further work is needed to overcome the problems of gas flow distribution and substrate shrinkage in large reactor. These two problems will affect the porosity of the matrix, and thus affect mass transfer, heat transfer, and microbial metabolism [73]. Jung Finkler et al. [74] constructed a packed bed bioreactor axial heat transfer model and calculated the heat generation dynamics based on the oxygen uptake rate. They found that the key to scale-up is to reduce the inlet temperature during the peak of heat generation. These studies will help to scale up the packed bed SSF bioreactor into a large-scale industrial production equipment. Wang et al. [75] used the Logistic equation, Luedeking-Piret equation, and Leudeking-Piret-like equation to describe microbial growth (R^2^ = 0.83), substrate consumption (R^2^ = 0.996), and product generation (R^2^ = 0.994) in a SSF system for producing fuel ethanol, achieving a relatively accurate description and prediction of fermentation process parameters. The study was conducted in conical flask, and an industrial-scale rotary drum SSF equipment was subsequently designed based on the model, achieving mechanized production and automated control of SSF fuel ethanol [76].

**Figure 3:**
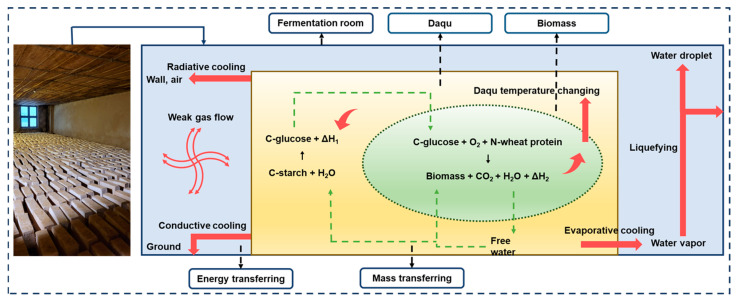
Heat balance analysis of the Daqu fermentation process in fermentation room. Reprinted/adapted with permission from Ref. [77] (2024, *Food and Fermentation Industries*).

The SSF mechanism of Baijiu is complex and the process is difficult to control. During the Daqu fermentation process, Zhao et al. [77] constructed a heat balance model to analyze the heat transfer in Daqu fermentation from an engineering perspective (Figure 3), and realized laboratory-scale Daqu fermentation virtual simulation and control [78], thus advancing the process of intelligent Daqu fermentation. Based on hyperspectral imaging technology, Jiang et al. [79] constructed a Back pigmentation neural network model to achieve rapid and accurate detection of the total acid content of Daqu, achieving rapid detection, which can be used to guide Daqu production.

In SSF, algebraic equation models such as fermentation kinetics and heat and mass transfer can reflect the internal mechanism relationship of the fermentation system and are easy to optimize the elementary model. However, the establishment of the current mechanism model is based on the assumption of independence of local laws, so there may be deviations when making large-scale predictions. Moreover, it is very difficult to determine the values of the kinetic parameters and how the structure of these models may even imply the impossibility of determining these values unequivocally. Statistical models such as black propagation neural networks have strong nonlinear fitting capabilities and do not require the establishment of mathematical models, but they cannot express the process mechanism of the fermentation system. In addition, they need to process massive data and conduct training and learning through large amounts of data. For different systems and different research objectives, the best adaptation model can be established (Table 3).

#### 3.3.2. Equipment Innovation

Fermentation equipment innovation is the key to overcoming the difficulties in collecting process parameters. Designing integrated equipment with parameter collection and control functions to achieve rapid and non-destructive monitoring of fermentation process parameters will be more conducive to the regulation and optimization of the fermentation process [80]. Li et al. [81] designed a new pilot fermentation tank (3 m^3^) and carried out temperature-controlled fermentation of Zhimaxiangxing Baijiu in the factory. By comparing the quality of crude Baijiu produced in the fermentation tank with that in the traditional pit, they found that the quality of crude Baijiu produced in the fermentation tank was significantly improved, and the yield was increased by 9.5%. Yue et al. [82] designed a laboratory-scale artificial pit (250 L) with a gas collection device inside, which not only allowed online observation of gas production changes, but also maintained a certain pressure in the pit. The equipment was heated and cooled using circulating water, replacing the uncontrollable natural geothermal system. This made Baijiu pit fermentation less susceptible to weather influences and enabled better monitoring and control.

In order to improve the intensive level of SSF of vinegar, Wang et al. [83] designed a rotary drum bioreactor that integrates the processes of feeding, discharging, inoculation, fermentation, vinegar pouring and vinegar smoking, thus improving the level of mechanized production. Compared with the traditional process, the new reactor has achieved automatic temperature control and reduced labor intensity. However, during the scale-up and industrial application research, it was found that when the scale-up was from 3 m^3^ to 36 m^3^, the output decreased and the production efficiency decreased [7]. Therefore, it is necessary to further optimize the process and promote the mechanization upgrade of the vinegar industry.

Compared with the Baijiu and vinegar industries, Chinese soy sauce industry has a higher degree of mechanization [84], but there is still a gap compared with developed countries. The development of new equipment can not only improve the quality of soy sauce, but also reduce costs. With the continuous innovation of fermentation equipment, the use of industrial large-scale fermentation tanks to replace traditional fermentation pond has become a production trend in the soy sauce industry. The scale of the fermentation tank can reach 200 tons [85]. It is also equipped with monitoring and control systems, with a high degree of automation, which reduces production costs [86].Zhang et al. uses a microfiber near-infrared spectrometer to monitor the digestibility of steamed soybeans, enzyme activity and formaldehyde content in real time, ensuring the high-quality production of soy sauce [87].

## 4. Prospects

**Figure 4:**
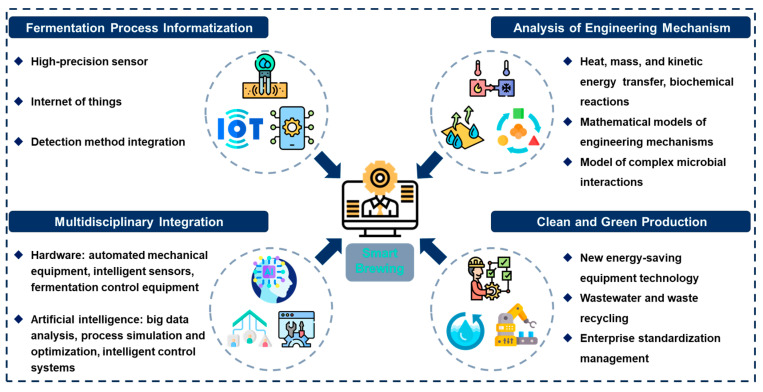
Future development trends of traditional Chinese SSF foods.

Due to the heterogeneity of the SSF system and the complex microbiota, there are still many difficulties in the mechanization, digitization, and intelligent transformation and upgrading of traditional Chinese solid-state fermented foods. Based on the development direction of traditional SSF process optimization and control, scale-up, intelligent fermentation, and clean production, analyzing the engineering mechanism of SSF process is the basis for achieving this goal. In order to promote the healthy and sustainable development of traditional Chinese solid-state fermented food industry and keep up with the pace of the times, the following problems and challenges need to be solved urgently (Figure 4).

(1) Fermentation process informatization: The acquisition of process parameters is the basis of all development. Traditional offline detection cannot provide timely feedback on the production process, and the data lacks continuity. The development of sensor technology and the Internet of Things provides technical support for online monitoring, but it is also necessary to design methods to integrate data from each section to realize the informatization of the entire process, and then use big data analysis to help understand and optimize the fermentation process. However, the resulting surge in data has put forward higher requirements on the information facilities. In addition, the SSF system is complex, and the sensors are easily affected by factors such as temperature, humidity, and microbial metabolism. Therefore, in order to achieve comprehensive application and promotion, it is necessary to overcome technical complexity.

(2) Analysis of engineering mechanisms: Most of the existing SSF process optimization solutions are based on direct empirical transformation, without involving engineering mechanisms, and are difficult to scale up and promote. Heat transfer, mass transfer, kinetic energy transfer and biochemical reactions are the focus of SSF process engineering research, which are not only directly related to microbial growth and metabolism, but also determine product quality. Establishing an engineering model to analyze the fermentation mechanism of traditional processes is the key to designing reactors and process control solutions.

(3) Multidisciplinary integration: The intelligent upgrade of the traditional SSF industry is a complex and systematic project, which requires precise control and efficient operation of hardware equipment, as well as intelligent analysis and optimization of software systems, that is, combined with artificial intelligence. Hardware: automated mechanical equipment, intelligent sensors, fermentation environment control equipment, etc.; software: big data analysis, process simulation and optimization, intelligent control systems, etc. To establish an intelligent, efficient and stable fermentation system, multidisciplinary integration is essential, especially the coordinated development of information technology, mechanical engineering, bioengineering and materials science.

(4) Clean and green production: During the transformation stage of traditional industries, clean production is also the key to maintaining healthy and sustainable development. Waste, wastewater, energy consumption, and improper production environment control during the fermentation process will not only cause environmental pollution, but also affect food quality and safety. Waste resource utilization, wastewater treatment technology, new equipment energy-saving technology, and standardized production environment management are also areas that the traditional food industry needs to pay attention to.

## 5. Conclusions

At present, traditional Chinese SSF industry, especially the Baijiu industry, is still in the early stages of transformation and upgrading. Not only do some traditional technical bottlenecks need to be broken through, but intelligent fermentation and clean production are also imminent. Based on the above analysis, it can be seen that traditional SSF has achieved rapid development in the field of engineering research in recent years, such as raw material processing and application research, analysis of hydrodynamics and heat transfer mechanisms of fermentation processes, process parameter optimization, establishment of fermentation process models and effect verification, as well as improvement and innovation of SSF reactors. traditional Chinese SSF food industry is actively seeking reform and development. With the continuous advancement of technology and the deep integration of multiple disciplines, it will usher in a new period of development with more intelligence, cleanliness and efficiency in the future.

## Figures and Tables

**Table 3:** Establishment and verification of SSF process mathematical model.

Model	Objective	Bioreactor	Reference
Product inhibition model	Predicting whether the system will overheat under different fermentation conditions	Fermentation jar	[71]
Two-phase two-dimensional model	Predicting the maximum temperature and packed bed height of the scale-up system	Packed bed bioreactor	[72]
Thermogenesis kinetic model	Predicting metabolic heat production	Packed bed bioreactor	[74]
Logistic equation, Luedeking equation, Leudeking-Piret-like equation	Predicting microbial growth, substrate consumption, and product generation during fermentation	Conical flask	[75]
		Rotary drum bioreactor	[76]
Heat balance model	Predicting Daqu temperature	Fermentation room	[77]
Black propagation neural network model	Predicting Daqu total acid	Fermentation room	[79]

## Data Availability

Data sharing not applicable. No new data were created or analyzed in this study.
